# New participant stratification and combination of urinary biomarkers and confounders could improve diagnostic accuracy for overactive bladder

**DOI:** 10.1038/s41598-020-59973-6

**Published:** 2020-02-20

**Authors:** Sepinoud Firouzmand, Ladan Ajori, John S. Young

**Affiliations:** 1 School of Pharmacy & Biomedical Sciences, University of Portsmouth, St. Michael’s Building, White Swan Road, Portsmouth, UK, PO1 2DT England, UK; 2 Shahid Beheshti University of Medical Sciences, Tehran, Iran; 3 Shohada-e-Tajrish Hospital, Tehran, Iran

**Keywords:** Diagnostic markers, Bladder

## Abstract

Overactive bladder (OAB) is a highly prevalent symptom complex characterised by symptoms of urinary urgency, increased frequency, nocturia, with or without urge incontinence; in the absence of proven infection or other obvious pathology. The underlying pathophysiology of idiopathic OAB is not clearly known and the existence of several phenotypes has been proposed. Current diagnostic approaches are based on discordant measures, suffer from subjectivity and are incapable of detecting the proposed OAB phenotypes. In this study, cluster analysis was used as an objective approach for phenotyping participants based on their OAB characteristic symptoms and led to the identification of a low OAB symptomatic score group (cluster 1) and a high OAB symptomatic score group (cluster 2). Furthermore, the ability of several potential OAB urinary biomarkers including ATP, ACh, nitrite, MCP-1 and IL-5 and participants’ confounders, age and gender, in predicting the identified high OAB symptomatic score group was assessed. A combination of urinary ATP and IL-5 plus age and gender was shown to have clinically acceptable and improved diagnostic accuracy compared to urodynamically-observed detrusor overactivity. Therefore, this study provides the foundation for the development of novel non-invasive diagnostic tools for OAB phenotypes that may lead to personalised treatment.

## Introduction

Idiopathic overactive bladder (OAB) is defined by the presence of bothersome symptoms of urinary urgency, increased frequency, nocturia, with or without urge incontinence in the absence of proven infection or other obvious pathology^1^. The prevalence of OAB ranges between 9% to 43% and 7% to 27% in female and male, respectively^2^.

The initial assessment of OAB includes documentation of OAB signs and symptoms, and exclusion of other diseases that present with some overlapping symptoms^2^. OAB studies to date have used varying combinations and/or severity of the main four OAB characteristic symptoms to identify patients for OAB studies’ inclusion and exclusion criteria. These differences across studies are considered as a challenge^2^ for interpretation, replication or clinical implementation of observed findings from these studies. This has resulted in attrition of well-needed new diagnostic methods such as many unsuccessful OAB-specific biomarker studies, and new drug development studies. Recently amended American Urological Association/Society of Urodynamics, Female Pelvic Medicine and Urogenital Reconstruction (AUA/SUFU) Guidelines on non-neurogenic OAB diagnosis and treatment^2^ highlight the need for better OAB stratification and the use of validated standardised measures for reporting subjective outcomes in clinical studies. Therefore, there is an unmet need for an objective OAB symptom-based classification approach.

Cluster analysis is a statistical approach used to reveal natural groupings within a population and to categorise individuals with similar characteristics into meaningful clusters. It has been used as such for the objective stratification of complex medical^3^ and psychiatric disorders^4^. As OAB is a symptom complex, subjecting its characteristic symptoms to cluster analysis may be used as an objective approach for participant stratification, but also may reveal the existence of groups of participants with specific combination and/or severity of symptoms (i.e. phenotypes of OAB^5^) that otherwise are not detectable using subjective human-based symptom analysis. Therefore, in this study, cluster-analysis was applied to data collected on the four main OAB characteristic symptoms and associated bothersome scores in order to assess the ability of such analysis in objectively identifying any meaningful groupings amongst participants.

Furthermore, the need for identification and development of OAB-specific biomarker(s) was another criterion highlighted in AUA/SUFU guidelines^2^. From the studies to date, the urinary levels of several biomarkers have shown to be altered in OAB patients^6^. The main drawback of these studies is a lack of sensitivity and specificity^6^. Previous attempts to elucidate urinary biomarkers for OAB have used univariate statistical analyses; thus neglecting many influencing variables and factors, such as the probable synergistic effects of combining biomarkers and confounders. The prevalence^7^ and severity^8^ of OAB increases with age. Some studies have reported its higher prevalence in female than male^9,10^, whereas others found no difference across genders^7,11^. Therefore, the impact of confounders such as age and gender is worthy of consideration in biomarker discovery analysis. Hence, monitoring a combination of biomarkers and participants’ confounders may produce tools with improved diagnostic accuracy. Therefore, in this study, we assessed the individual and combinational abilities of six candidate OAB urinary biomarkers plus participants’ confounders (including age and gender) in predicting groups identified using the new symptom-based OAB classification approach. These biomarkers included adenosine triphosphate (ATP)^12^; acetylcholine (ACh)^13^; nitric oxide (NO)^14^ and its oxidation product, nitrite; interleukin-5 (IL-5); and monocyte chemoattractant protein 1 (MCP-1)^15^). The six chemical biomarkers were chosen because previous research had shown their changes in association with OAB.

Overall, this study aimed to better stratify patients based on OAB symptoms and subsequently characterise stratified groups based on urinary biomarkers.

## Results

### Participants

**Figure 1:**
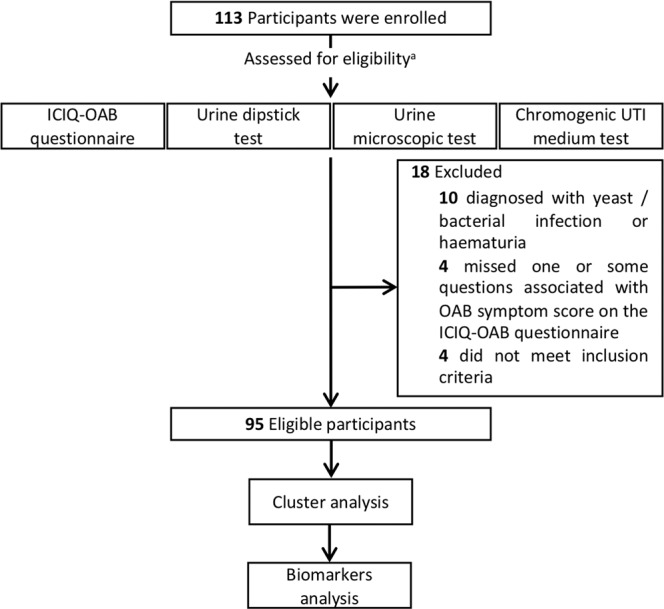
Flow diagram of participants’ selection, subsequent tests and analysis performed. ICIQ-OAB = International consultation on incontinence questionnaire - overactive bladder. ^a^see Methods and Materials section for inclusion and exclusion criteria.

This study and all its procedures were approved by the National Research Ethics Service (NRES) Committee South Central Berkshire (REC reference: 13/SC/0501). All research was performed in accordance with procedures and regulations at the University of Portsmouth. Written informed consent was obtained from all participants. According to the performed power analysis, a minimum of 84 participants was required to provide an 80% power to detect a correlation of 0.3 between the symptom scores and each urine biomarker at 5% two-sided significance level. One hundred and thirteen self-selecting volunteers with or without symptoms of OAB were recruited to allow for those excluded according to the inclusion/exclusion criteria and for a number of drop-outs. Amongst 113 recruited participants, ten were diagnosed with yeast/bacterial infection or haematuria; four participants failed to complete one or some of the questions associated with the main four OAB characteristic symptoms on the ICIQ-OAB questionnaire and four did not meet the inclusion criteria and therefore excluded. Hence, 95 participants were eligible to be involved in the further analyses (Fig. 1).

### Distribution of OAB characteristic symptom and bothersome scores

**Figure 2:**
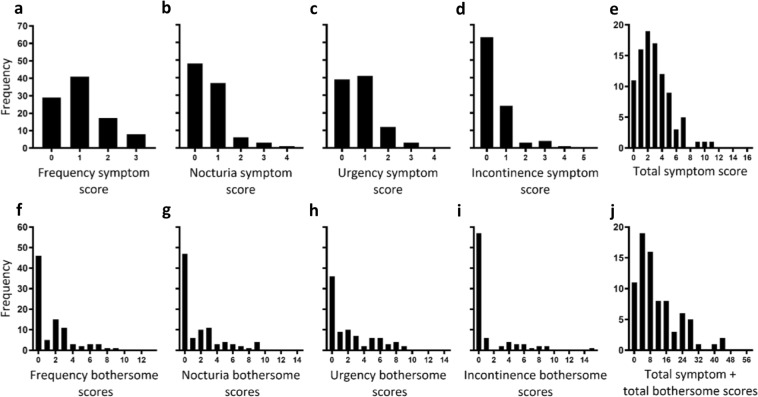
Frequency distributions of ICIQ-OAB urinary symptom scores and associated bothersome scores of eligible participants. (**a–e**) Frequency distributions (n = 95) of OAB characteristic symptom scores of eligible participants. (**f–j**) Frequency distributions (n = 81) of OAB symptom associated bothersome scores of eligible participants. 14 participants left one or some of the symptom associated bothersome questions blank. See Supplementary Table 1 for ICIQ-OAB questionnaire.

The frequency distributions of eligible participants’ OAB characteristic symptoms and associated bothersome scores are shown in Fig. 2a–e,f–j, respectively. Distributions were right-skewed, where no apparent bimodal or multimodal distributions suggestive of distinct groupings (e.g. suggestive of OAB phenotypes and asymptomatics) was noticeable.

### Phenotyping participants based on their reported profile of OAB characteristics using cluster analysis

Two-step cluster analysis was used to identify any natural groupings amongst participants’ OAB related characteristics including symptom scores, symptom associated bothersome scores and symptom plus associated bothersome scores (Supplementary Table S2).

**Figure 3:**
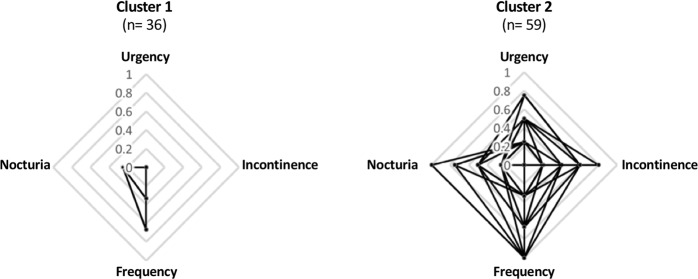
Radar plot of the distributions of the participants’ OAB characteristic symptom scores amongst the identified clusters. The distribution of participants’ responses to each OAB symptom question was range standardised on a 0–1 scale were 0 represents the lowest and 1 represents the highest symptom severity for each OAB characteristic symptom, i.e. the centre of the plots represents the lowest symptom scores and the outermost layer of the plots represents the highest symptom scores. See Table 1 for the measures of central tendency (i.e. median) of each OAB characteristic symptom in each cluster.

In general, two natural groups were identified based on all the different aspects of the participants’ OAB associated characteristics (Supplementary Table S2). Fourteen participants failed to complete one or some bothersome associated questions, therefore, groups identified based on participants’ OAB characteristic symptom scores only (n = 95) were chosen for further analyses. Amongst 95 participants, 36 and 59 participants were assigned by the cluster analysis to clusters 1 and 2, respectively (Table 1). Urgency was identified as the main cluster predictor component and was followed by incontinence, frequency and nocturia, in order of importance (Supplementary Table S2). In other words, urgency (the key OAB symptom^16^) was the primary factor accounting for the differences between the two groups. The distribution of urinary symptom scores amongst the two identified clusters is reported in Table 1 and depicted in Fig. 3. No significant differences were observed between the proportion of male and female between the two groups (Table 1). Participants in cluster 2 had statistically significantly higher urgency (*p* ≤ 0.0001); incontinence (*p* ≤ 0.0001); frequency (*p* = 0.0048) and nocturia (*p* = 0.0115) scores and were significantly older (*p* = 0.0079, male participants in both clusters were significantly older than their female counterparts (data not shown)) compared to those in cluster 1 (Table 1). Not all the participants in cluster 1 were truly asymptomatic; that is, with a symptom score of zero for all the four OAB-associated symptoms. This was expected as the recruitment of a truly asymptomatic age-matched (i.e. 22–93 yrs) group is challenging given the prevalence of lower urinary tract symptoms in nearly two-thirds of adults^7^. Therefore, subsequent analyses were based on the identified groups, i.e. low OAB symptomatic score group (cluster 1) and high OAB symptomatic score (cluster 2).

**Table 1:** Description of clusters identified using Two-step cluster analysis.

Cluster description	Cluster 1	Cluster 2	*p* value
n	36	59	
Gender (F/M)	20/16	41/18	ns^a^
Age, mean (range) (yrs)	49 (21–90)^b^	56 (22–93)	0.0079^c^
Urgency^d^, median (IQR)	0 (0.00–0.00)	0.25 (0.25–0.37)	≤0.0001^c^
Incontinence^d^, median (IQR)	0.0 (0.00–0.00)	0.20 (0.00–0.20)	≤0.0001^c^
Frequency^d^, median (IQR)	0.33 (0.00–0.33)	0.33 (0.33–0.67)	0.0048^c^
Nocturia^d^, median (IQR)	0.00 (0.00–0.25)	0.25 (0.00–0.25)	0.0115^c^

n = number of participants in each cluster; ns = not significant; IQR = Interquartile range, 1^st^ quartile-3^rd^ quartile.

^a^Z-test was used for comparison.

^b^One missing age value, n = 35 for cluster 1.

^c^Mann-Whitney test.

^d^Symptoms scores were range standardised on a 0 to 1 scale.

### Comparison of the urinary concentrations of the individual biomarkers between the two identified groups

A comparison of the urinary concentrations of each biomarker between the two identified groups is reported in Supplementary Fig. 1. There was no statistically significant difference in the levels of individual urinary biomarkers between the two identified groups. This may be due to the fact that there are changes to biomarker levels already in the low OAB symptomatic score group (cluster 1) compared to urine from truly asymptomatic participants, and these biomarkers do not change much more as symptoms become more severe. Alternatively, because our comparison is not between two extreme ends of the OAB symptom severity spectrum (i.e. asymptomatic vs. severe OAB), differences in individual associated biomarker concentrations in our comparison are small and not statistically different. Therefore, such biomarkers - used individually - would not be suitable to identify initial stages of OAB development.

### The ability of urinary biomarkers and confounders in predicting the high OAB symptomatic score group from the low OAB symptomatic score group

The individual ability of the candidate urinary biomarkers (including ATP, ACh, NO, Nitrite, MCP-1 and IL-5), and confounders (age and gender) in predicting the identified high OAB symptomatic score group (cluster 2) from the low OAB symptomatic score group (cluster 1) was assessed using binary logistic regression (Supplementary Table S3). Age was the only parameter that was shown to have statistically significant prediction power (*p* = 0.041, Supplementary Table S3). In order to assess whether the addition of other parameters would increase the prediction power of age, 23 combination models were developed by incorporating candidate urinary biomarkers and gender (Supplementary Table S3). Amongst all the developed logistic models, seven models including combination 1 (age + gender, *p* = 0.020); combination 10 (age + gender + Il-5, *p* = 0.011); combination 12 (age + gender + ACh, *p* = 0.039); combination 14 (age + gender + IL-5 + ACh, *p* = 0.015); combination 15 (age + gender + IL-5 + ACh + ATP, *p* = 0.045); combination 17 (age + gender + IL-5 + ATP, *p* = 0.026) and combination 18 (age + gender + IL-5 + NO, *p* = 0.024) were also shown to have statistically significant prediction powers (Supplementary Table S3). All the developed models passed goodness of fit test (Hosmer-Lemeshow test in Supplementary Table S3).

**Figure 4:**
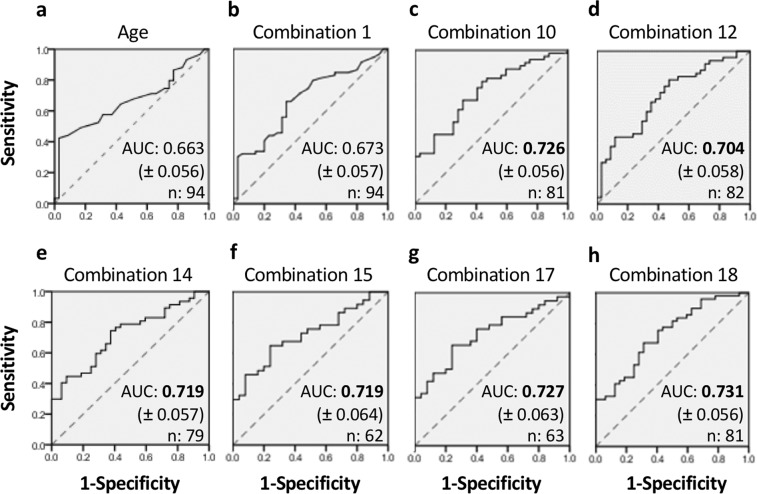
Receiver-operating characteristic curves (ROCs). (**a**) Age; (**b**) Combination 1 = Age + Gender; (**c**) Combination 10 = Age + Gender + IL-5; (**d**) Combination 12 = Age + Gender + ACh; (**e**) Combination 14 = Age + Gender + IL-5 + ACh; (**f**) Combination 15 = Age + Gender + IL-5 + ACh + ATP; (**g**) Combination 17 = Age + Gender + IL-5 + ATP; (**h**) Combination 18 = Age + Gender + IL-5 + NO. AUC = Area under the ROC curve; Value in parenthesis = standard error; Bold value = 0.7 ≤ AUC < 0.8 meaning predictive model has clinically acceptable discriminatory power; Solid line = prediction model curve; Grey diagonal dashed line = chance line.

The diagnostic accuracy of the developed logistic models in distinguishing the high OAB symptomatic score group (cluster 2) from the low OAB symptomatic score group (cluster 1) was assessed by ROC analysis (Fig. 4). Amongst the seven combination models, only six models (i.e. combinations 10; 12; 14; 15; 17 & 18) were shown to have clinically acceptable diagnostic powers (i.e. 0.7 ≤ AUC < 0.8, Fig. 4). Age, despite having a statistically significant prediction power, did not have clinically acceptable diagnostic power (AUC = 0.663, Fig. 4). Age and gender combined have an AUC of 0.670 with a sensitivity and specificity both of 66%. ROC curves for the logistic models without statistically significant prediction powers are also shown in Supplementary Fig. 2.

Furthermore, the performance (i.e. positive predictive value (PPV) and negative predictive value (NPV)) of the six combination models that were shown to have clinically acceptable diagnostic powers were assessed based on the prevalence range of OAB in female (between 9% to 43%) and male (between 7% to 27%)^2^ and was compared to urodynamically-observed detrusor overactivity (DO) (Table 2). All the six logistic models were shown to have higher NPV and PPV values compared to DO at both lower and higher end of the prevalence range for both female and male (Table 2).

**Table 2:** Clinical performance of the constructed OAB predictive models based on the prevalence range of OAB in female and male.

	Based on maximum Youden Index (*J*)			Based on prevalence range of OAB in female				Based on prevalence range of OAB in male			
				Lower end 9%		Top end 43%		Lower end 7%		Top end 27%	
Predictive model	*P*_cluster 2_ cut-off	Sensitivity (%)	Specificity (%)	PPV (%)	NPV (%)	PPV (%)	NPV (%)	PPV (%)	NPV (%)	PPV (%)	NPV (%)
Combination 10	0.51	67	69	18	96	62	74	14	97	44	85
Combination 12	0.46	81	53	15	97	57	79	12	97	39	88
Combination 14	0.46	74	63	16	96	60	76	13	97	42	87
Combination 15	0.56	65	76	21	96	67	74	17	97	50	85
Combination 17	0.56	66	76	21	96	67	75	17	97	50	86
Combination 18	0.51	67	69	18	96	62	74	14	97	44	85
Urodynamic DO^a^		54	68	14	94	56	66	11	95	38	80

*P*_cluster 2_ = Probability of an individual being in the identified high OAB symptomatic score group; PPV = Positive predictive value; NPV = Negative predictive value.

^a^Sensitivity and specificity values for urodynamic test was obtained from Digesu *et al*.^23^ study where the presence of DO was used as a marker for diagnosing those presenting with OAB symptoms.

### Predicting the probability of an individual being in the identified high OAB symptomatic score group

Combination 17 was shown to have the highest PPV (21%, 67% for female and 17%, 50% for male) and NPV (96%, 75% for female and 97%, 86% for male) values compared to the other logistic models or urodynamic DO (Table 2), therefore, an equation predicting the likelihood of the identified high OAB symptomatic score group (cluster 2) was constructed based on the combination 17 (Table 3). Henceforth, by measuring urinary levels of IL-5 and ATP and entering their creatinine-normalised values plus values for age and gender in the formula, the probability of an individual being in the identified high OAB symptomatic score group (*p*_cluster 2_) could be calculated.

**Table 3:** OAB predictive equation.

Predictive model	OAB prediction equation
Combination 17	Probability of an individual being in the high OAB symptomatic score group (*p*_cluster 2_) = 1/1 + e^−X^, where X = (−3.090) + 5.393 x subject’s age^b^ + 1.797 x Gender (Female = 1, Male = 0) + 34.767 × [IL-5/Cr]^b^ + (−562.743) x [ATP/Cr]^b^

e = exponential-e; Cr = Creatinine, urinary biomarker value needs to be normalised to urinary creatinine concentration before being entered in the equation.

^b^Variable(s) needs to be range standardised before being entered into the equation, age to 120 yrs old, IL-5/Cr to 100 and ATP/Cr to 0.000001.

Standard errors (SE) for constants and coefficients are reported in Table S4.

## Discussion

OAB is a symptom complex that may significantly impact sufferers’ quality of life including their physical and mental health, social, sexual, economical and professional lives^17,18^. Idiopathic OAB is a symptom-based diagnosis, however, a consensus regarding the symptom severity or combination necessary for accurate OAB diagnosis is lacking. AUA/SUFU identified that inconsistent combinations of OAB symptoms used as inclusion criteria across studies are challenging for interpretation, and they have recommended better stratification of OAB and phenotyping of its characteristic symptoms^2^. In this study, we aimed to objectively stratify patients based on OAB symptoms and subsequently characterise stratified groups based on urinary biomarkers.

In order to stratify, cluster analysis was used to objectively phenotype participants based on their reported profile of OAB characteristic symptoms and/or associated bothersome scores. Despite programming the statistical analysis to detect the existence of a maximum number of 15 groups, only two groups were identified, where one had statistically significantly higher OAB characteristic symptom scores compared to the other one. Urgency was the main factor accounting for differences between the two identified groups, followed by incontinence, frequency and nocturia. Previous studies, such as Cinar *et al*.^19^ and Hall *et al*.^20^, took the similar approach for clustering participants based on their urological symptoms, however, these studies were based on a broad range of lower urinary tract symptoms, rather than focusing on symptoms associated with a distinct type of urological disease/symptom complex. Peyronnet *et al*. 2019 suggested the existence of several OAB phenotypes based on the different underlying mechanisms and pathophysiological cofactors^5^. Therefore, understanding the changes that occur across different phenotypes of OAB requires an approach that can identify such subpopulations at early developmental stages in order to enable researchers to monitor temporal pathophysiological changes. To the best of authors’ knowledge, this is the first study that has applied such an objective approach to identify OAB phenotypes. In addition, in order to increase the sensitivity of such approach in detecting changes that occur at the initial stages across OAB phenotypes, participants were selected from those that were expected to have mild to moderate OAB characteristic symptoms. In other words, the involvement of those participants at the higher end of the OAB symptom spectrum was kept to a minimum, in order to avoid their potential effect on cluster analysis through masking the identification of potential indiscernible changes that occur at the initial developmental stages across OAB-phenotypes. Future studies should involve those across the entire range of the OAB symptom spectrum.

Exclusion of other diseases that present some overlapping symptoms with OAB is part of the initial OAB diagnostic process. In this study, this was achieved by having detailed exclusion criteria (see Methods and Materials) and through the use of a comprehensive range of tests to exclude UTI including microscopic examination, dipstick urinalysis and chromogenic UTI medium test. According to AUA/SUF, the use of urine culture as part of the routine urinalysis for OAB is not necessary unless indication of infection is found during dipstick or microscopic examination, or may be performed at the discretion of the clinician^2^. However, due to the deficiencies in UTI diagnostic methods^21^ and as UTI is considered as the most commonly misdiagnosed condition among women with OAB^22^, all the participants in this study were subjected to a combination of tests to ensure UTI exclusion.

However, limitations of the current stratification method deserve mention. Similar to the current initial subjective OAB classification, the current objective participant stratification method was based on participants’ self-reported symptoms, meaning diagnosis is based on subjective measures. The use of other diagnostic measures, such as urodynamics, are not recommended by AUA/SUFU in the initial diagnostic workup of uncomplicated OAB^2^. In addition, the association between invasive means of urodynamically-demonstrable detrusor overactivity (DO) and OAB symptoms remains questionable, with half of patients with OAB symptoms not exhibiting DO, whereas DO was observed in two-thirds of those without any OAB symptoms^23–25^. Furthermore, according to the recently published European Association of Urology guidelines on assessment and nonsurgical management of urinary incontinence (UI), the evidence of clinical benefit of urodynamically-observed DO with the UI treatment outcome remains inconsistent^26^. Therefore, non-invasive surrogates such as urinary biomarkers, if proven to be sensitive and specific for OAB-phenotypes, might be the key to solve the subjectivity issues associated with OAB diagnosis.

Following the identification of two distinct OAB symptom-associated groups, we assessed the ability of several potential OAB urinary biomarkers including ATP, ACh, nitrite, MCP-1 and IL-5 and participants’ confounders such as age and gender, in differentiating the high OAB symptomatic score group from the low OAB symptomatic score group. Previous discovery studies based on a single putative OAB biomarker have suffered from issues of sensitivity and specificity^27^, perhaps attributable to the limitations of univariate statistical approaches i.e. each biomarker was considered discretely and independent of participants’ confounders or other biomarkers^28^. Multivariate statistical approaches were used in this study to explore the synergistic effects of combining confounders and candidate biomarkers as a discriminatory means for the identified high OAB symptomatic score group (cluster 2). Twenty-three combination models, comprising confounders and urinary biomarkers, were developed; of which six models were shown to have both satisfactory prediction and diagnostic (i.e. sensitivity and specificity) abilities in distinguishing the high OAB symptomatic score group (cluster 2) from the low OAB symptomatic score group (cluster 1). Age and gender themselves are reasonable predictors of OAB; that is, they differentiate between the low OAB symptomatic score group (cluster 1) and the high OAB symptomatic score group (cluster 2) with an AUC of 0.67 and a sensitivity and specificity both of 66%. The diagnostic power of age and gender is not unexpected given a well-documented increase in OAB prevalence with age^7^ and a greater prevalence in females^9^. However, age and gender are confounders for different types of LUTS, therefore, a combination of age, gender and OAB biomarkers improve the ability to detect OAB. In this study the addition of urinary biomarkers improved the diagnostic power by up to 6% (i.e. to an AUC of up to 0.73; Table 2). In comparison to age and gender, some combinations are more sensitive (e.g. Combination 12, 81% sensitivity; Table 2) and others more specific (e.g. Combination 17, 76% sensitivity; Table 2). More work is required to refine the biomarker combinations in order to achieve an improved diagnostic power. To the best of authors’ knowledge, the current study has for the first time assessed the predictability and discriminatory powers of the combination of potential urinary biomarkers and participants’ confounders for OAB using multivariate statistical methods. Peyronnet *et al*. 2019 suggested that OAB phenotypes are possibly not exclusive and are likely to overlap, which may explain the reason behind success of some combination therapies for some OAB patients^5^. The same rationale could be true in the case of the diagnosis of OAB phenotypes, where a single biomarker could not have the adequate discriminatory power, therefore, a combination of several parameters could improve diagnostic accuracy.

As no gold standard OAB diagnostic method is currently available, the clinical performance of the six combination models was compared to urodynamically-observed DO. All the six combination models were shown to have better performance compared to urodynamically-observed DO. Amongst these combination models, combination 17, i.e. a combination of age, gender, ATP and IL-5, was shown to have the highest PPV and NPV values, and were similar to the performance of urine-based tests for other diseases such as bladder carcinoma^29^ or prostate cancer^30^. An equation based on combination 17 was constructed to predict the likelihood of the identified high OAB symptomatic score group. By measuring the urinary levels of IL-5 and ATP and entering their creatinine-normalised values, in addition to age and gender, in to the formula, the possibility of an individual being in the identified high OAB symptomatic score group could be predicted. This study provides the foundation for the development of novel non-invasive diagnostic tools for OAB, where the effects of biomarkers and key OAB confounders^31^ could be considered in conjunction.

### Limitations

By ruling out similarly-presenting conditions at the point of recruitment (see ‘Exclusion criteria’) and by performing urine pathology testing, we had hoped to recruit participants with a spectrum of lower urinary tract symptoms attributable to OAB. We acknowledge, however, that the ICIQ-OAB questionnaire lacks the ability to differentiate between different types of incontinence such as urge urinary incontinence, mixed urinary incontinence or stress incontinence. Although all the participants in this study with incontinence (i.e. an incontinence score of >0) had other OAB characteristic symptoms, more needs to be done in the future to differentiate between different types of incontinence. Furthermore, we cannot rule out the possibility that OAB was secondary to outflow obstruction. Future studies should therefore either involve participants with a broad range of OAB symptoms in order to allow the identification of further OAB phenotypes, and should be extended to include more participants and those with mixed urinary incontinence or, alternatively, include more rigorous recruitment criteria to exclude other possible overlapping conditions such as stress incontinence and polyuria. In addition, the involvement of other potential urinary biomarkers such as nerve growth factor^32^ and brain-derived neurotrophic factor^33^ and other OAB influencing factors such as body mass index^34^, smoking and alcohol intake should be considered. Furthermore, the clinical utility and performance of prediction algorithms should be tested in large, longitudinal studies.

## Conclusion

This study aimed to address the need for better OAB stratification. By using cluster analysis, we objectively phenotyped participants based on their urinary symptoms. This approach could be used in future to better stratify OAB phenotypes. Furthermore, the identification of combinations of urinary biomarkers that are associated with, and unique to, our stratified groups, provides the means to identify those phenotypes. We focus on one combination (comprising biomarkers and confounders) that together have improved diagnostic accuracy (sensitivity and specificity) and clinical performance (positive and negative predictive values) for OAB compared to the current available methods. When tested in large, longitudinal studies, this approach and its findings offer the potential for the development of more accurate and non-invasive diagnostic tools.

## Methods and Materials

### Recruitment of participants

This study and all its procedures were approved by the National Research Ethics Service (NRES) Committee South Central Berkshire (REC reference: 13/SC/0501). All methods were carried out in accordance with the guidelines and regulations of the University of Portsmouth. Written informed consent was obtained from all participants. Power analysis was performed in order to indicate the required number of participants for this study. A total of 113 volunteer participants (between 2014 and 2016) were recruited via volunteer sampling from the University of Portsmouth; the Briars Greensleeves Homes Trust-Isle of Wight; the National Federation of Women’s Institutes and Portsmouth Pensioners’ Association. Participants were asked to complete a validated OAB questionnaire (the International consultation on incontinence questionnaire - overactive bladder (ICIQ-OAB)) (Supplementary Table S1) and to provide a fresh midstream urine sample. Collected samples and data were made anonymous using an ID code system.

#### Inclusion criteria

Male or female participants aged ≥18 and able to give informed consent for participation in the study.

#### Exclusion criteria

Male or female participants aged ≤18; taking any medication for OAB; unable to give informed consent; diagnosed with neurologic disease (stroke, MS, Parkinson’s disease, spinal cord injury); have history of uterine, cervical, vaginal or urethral cancer; history of cyclophosphamide use or any type of chemical cystitis; history of benign or malignant bladder tumours; have had *Botulinum* toxin injections, neuromodulation or augmentation cystoplasty.

#### Urine pathology tests

Microscopic examination, dipstick urinalysis and chromogenic urinary tract infection (UTI) medium test were immediately performed on a small proportion of each collected urine sample. Any sample with positive test result was excluded from the study. The remainder of the urine sample was centrifuged (at 4000 rpm, 10 mins, at 4 °C), separated into cell pellet and supernatant and stored at −80 °C before being subjected to biomarker analyses.

### Biomarker assays

The urinary (cell-free) concentrations of the candidate biomarkers were measured using ENLITEN® ATP Assay System Bioluminescence Detection Kit (FF2000, Promega, UK); Amplex® Red Acetylcholine/Acetylcholinesterase assay (Invitrogen™ Molecular Probes™, A12217, UK); Sievers Nitric Oxide Analyser (NOA™ 280i, Analytix, UK); BD OptEIATM human MCP-1 enzyme-linked immunosorbent assay (ELISA) (559017, BD biosciences, UK); Quantikine® human IL-5 ELISA Kit (R&D Systems®, D5000B, UK) and the OptEIA^TM^ Human IL-5 ELISA Set (555202, BD biosciences, UK) according to the manufacturers’ instructions.

### Creatinine assay

Urinary creatinine (Cr) was measured using the Cayman Creatinine (urinary) Colourimetric Assay Kit (CAY500701, Cambridge Bioscience, UK), following the manufacturer’s instructions. All urinary biomarker values were normalised to corresponding Cr concentrations.

### Statistical analysis

#### Cluster analysis

TwoStep cluster analysis was performed using IBM SPSS statistics 22.0 on the range standardised (0–1 scale) ICIQ-OAB questionnaire data, including symptom scores; symptom associated bothersome scores and symptom plus associated bothersome scores. The software was programmed to automatically identify a maximum number of 15 clusters. Clusters formed based on symptom scores were chosen for further analyses due to the higher number of involved participants.

#### Binary logistic regression

The ability of the candidate biomarkers and participants’ confounders (age and gender), individually or in combination, in predicting the probability of an individual being in the identified high OAB symptomatic score group (cluster 2) was studied using binary logistic regression analysis (IBM SPSS statistics 22.0). Each variable was range standardised to the highest possible number that could be measured for any one human (and even for some biomarkers the considered value was much higher) i.e. age was range standardised to 120; ATP/Cr to 0.000001; ACh/Cr to 0.1; NO/Cr to 20000; Nitrite to 200; MCP-1/Cr to 100 and IL-5/Cr to 100. Therefore, any measured value in the future could be range standardised to the same values used in this study and consequently could be placed in the generated logit equation to estimate the probability of an individual being in the identified high OAB symptomatic score group (cluster 2).

#### Receiver Operating Characteristic (ROC), positive predictive value (PPV) and negative predictive value (NPV)

ROC curve analysis (IBM SPSS statistics 22.0) was used in order to evaluate the discriminatory power of the generated OAB prediction models using predicted probability values generated by logistic regression analyses. The following area under the curve (AUC) criteria was used to determine the discriminatory power of the generated predictive models: 0.5 ≤ AUC < 0.6: no discriminatory power; 0.6 ≤ AUC < 0.7: poor discriminatory power; 0.7 ≤ AUC < 0.8: acceptable discriminatory power; 0.8 ≤ AUC < 0.9: excellent discriminatory power and 0.9 ≤ AUC ≤ 1: outstanding discriminatory power^35^. The optimal cut-off values of the predicted probability of an individual being in the high OAB symptomatic score group (*p*_cluster 2_) for the selected predictive models were determined as the value with the maximum Youden Index (*J* = sensitivity + specificity – 1). PPV and NPV of each predictive model was calculated based on its sensitivity and specificity at the optimal cut-off value and based on the prevalence range of OAB in female (between 9% to 43%) and male (between 7% to 27%)^2^, and was compared to those for urodynamically-observed detrusor overactivity (DO) from Digesu *et al*. 2003 study^23^. ROC curve analysis was not performed as part of the Digesu *et al*. 2003 study, hence, data on the AUC and optimal cut-off values were not available.

#### Equation predicting the probability of an individual being in the identified high OAB symptomatic score group

An equation to predict the probability of an individual being in the identified high OAB symptomatic score group (cluster 2) was constructed based on the constant and coefficients values obtained from the logistic regression analysis for combination model 17.

## Supplementary information

Supplementary Dataset 1.

## Data Availability

The data that were generated and/or analysed for this study are available from the corresponding author upon request.
