# Supplementary material for: New participant stratification and combination of urinary biomarkers and confounders could improve diagnostic accuracy for overactive bladder
Source: Sci Rep. 2020 Feb 20;10:3085. doi: 10.1038/s41598-020-59973-6 (PMC7033236; doi:10.1038/s41598-020-59973-6)
Supplement: Supplementary file 1 — Supplementary Dataset 1. [file 41598_2020_59973_MOESM1_ESM.docx]

**New participant stratification and combination of urinary biomarkers and confounders could improve diagnostic accuracy for overactive bladder**

Sepinoud Firouzmand PhD^a^, Ladan Ajori MD^b,c^, John S. Young PhD^a,*^

^a^ School of Pharmacy & Biomedical Sciences, University of Portsmouth, St. Michael's Building, White Swan Road, Portsmouth, UK, PO1 2DT.

^b^ Shahid Beheshti University of Medical Sciences, Tehran, Iran.

^c^ Shohada-e-Tajrish Hospital, Tehran, Iran.

* Corresponding Author: John S. Young

Corresponding author’s contact details:

E: john.young@port.ac.uk

T: +44 (0)23 9284 3564

Address: School of Pharmacy and Biomedical Sciences, University of Portsmouth, St. Michael's Building, White Swan Road, Portsmouth, UK, PO1 2DT

**Table S1 – ICIQ-OAB question descriptions and standardised scales used in the Two-step cluster analysis.**

|  | ICIQ-OAB question | Answers | Scale | Range standardised scale |
| --- | --- | --- | --- | --- |
| Frequency | How often do you pass urine during the day? | Every four hours or more | 0 | 0.00 |
|  |  | Every three hours | 1 | 0.33 |
|  |  | Every two hours | 2 | 0.67 |
|  |  | Hourly | 3 | 1.00 |
| Nocturia | During the night, how many times do you have to get up (wake up) to urinate on average? | None | 0 | 0.00 |
|  |  | One time | 1 | 0.25 |
|  |  | Two times | 2 | 0.50 |
|  |  | Three times | 3 | 0.75 |
|  |  | Four or more times | 4 | 1.00 |
| Urgency | Do you have to rush to the toilet to urinate? | Never | 0 | 0.00 |
|  |  | Occasionally | 1 | 0.25 |
|  |  | Sometimes | 2 | 0.50 |
|  |  | Most of the time | 3 | 0.75 |
|  |  | All of the time | 4 | 1.00 |
| Incontinence | Does urine leak before you can get to the toilet? | Never | 0 | 0.00 |
|  |  | About once a week or less often | 1 | 0.20 |
|  |  | Two or three times a week | 2 | 0.40 |
|  |  | About once a day | 3 | 0.60 |
|  |  | Several times a day | 4 | 0.80 |
|  |  | All the time | 5 | 1.00 |

**Table S2 – Identified clusters via Two-step cluster analysis.**

| Cluster analysis component | n | NIC | Cluster size | Cluster predictor importance score |
| --- | --- | --- | --- | --- |
| Symptom scores | 95 | 2 | Group 1: 36 Group 2: 59 | Urgency: 1.00 Incontinence: 0.28 Frequency: 0.13 Nocturia: 0.11 |
| Bothersome scores | 81^a^ | 2 | Group 1: 51 Group 2: 30 | Urgency: 1.00 Incontinence: 0.65 Frequency: 0.42 Nocturia: 0.28 |
| Symptom + bothersome scores | 81^a^ | 2 | Group 1: 59 Group 2: 22 | Urgency: 1.00 Incontinence: 0.99 Frequency: 0.58 Nocturia: 0.38 |
| n= Number of participants involved in the cluster analysis; NIC= Number of identified clusters via cluster analysis; Cluster predictor importance score= 0 (weakest) – 1 (strongest) cluster predictor.  ^a^= 14 participants left one or some of questions associated with bothersome scores blank. | | | | |

**Table S3 – Prediction abilities of candidate biomarkers and participants’ confounders assessed individually and in combination, using Binary Logistic Regression.**

| Predictive model |  |  | Logistic Regression parameters | | | |
| --- | --- | --- | --- | --- | --- | --- |
|  | n | e | Pr  Null model (%) | Pr  New model (%) | O test  *(p* value) | HL test  *(p* value) |
| Gender | 95 | 1 | 62 | 62 | 0.171 | 0.000 |
| Age | 94 | 2 | 63 | 62 | **0.041** | 0.060 |
| Volume | 62 | 34 | 65 | 65 | 0.745 | 0.104 |
| ATP | 66 | 30 | 59 | 59 | 0.755 | 0.191 |
| ACh | 83 | 13 | 58 | 58 | 0.937 | 0.241 |
| NO | 85 | 11 | 59 | 59 | 0.773 | 0.454 |
| Nitrite | 83 | 13 | 59 | 59 | 0.254 | 0.163 |
| MCP-1 | 80 | 16 | 59 | 59 | 0.312 | 0.919 |
| IL-5 | 82 | 14 | 60 | 59 | 0.496 | 0.867 |
| Combination 1  Age, Gender | 94 | 2 | 63 | 67 | **0.020** | 0.283 |
| Combination 2  Age, Nitrite | 82 | 14 | 60 | 61 | 0.070 | 0.236 |
| Combination 3  Age, MCP-1 | 79 | 17 | 60 | 60 | 0.225 | 0.155 |
| Combination4  Age, IL-5 | 81 | 15 | 61 | 58 | 0.089 | 0.020 |
| Combination 5  Age, ATP | 66 | 30 | 59 | 59 | 0.285 | 0.117 |
| Combination 6  Age, ACh | 82 | 14 | 59 | 57 | 0.121 | 0.014 |
| Combination 7  Age, NO | 84 | 12 | 60 | 58 | 0.196 | 0.027 |
| Combination 8  Age, Gender, Nitrite | 82 | 14 | 60 | 68 | 0.064 | 0.705 |
| Combination 9  Age, Gender, MCP-1 | 79 | 17 | 60 | 62 | 0.061 | 0.021 |
| Combination 10  Age, Gender, IL-5 | 81 | 15 | 61 | 67 | **0.011** | 0.677 |
| Combination 11  Age, Gender, ATP | 66 | 30 | 59 | 67 | 0.059 | 0.216 |
| Combination 12  Age, Gender, ACh | 82 | 14 | 59 | 65 | **0.039** | 0.473 |
| Combination 13  Age, Gender, NO | 84 | 12 | 60 | 64 | 0.051 | 0.405 |
| Combination 14  Age, Gender, IL-5, ACh | 79 | 17 | 60 | 66 | **0.015** | 0.281 |
| Combination 15  Age, Gender, IL-5, ACh, ATP | 62 | 34 | 60 | 66 | **0.045** | 0.849 |
| Combination 16  Age, Gender, IL-5, ACh, ATP, NO | 62 | 34 | 60 | 66 | 0.079 | 0.736 |
| Combination 17  Age, Gender, IL-5, ATP | 63 | 33 | 60 | 66 | **0.026** | 0.726 |
| Combination 18  Age, Gender, IL-5, NO | 81 | 15 | 61 | 67 | **0.024** | 0.550 |
| Combination 19  Age, IL-5, ATP | 63 | 33 | 60 | 59 | 0.252 | 0.041 |
| Combination 20  Age, IL-5, NO | 81 | 15 | 61 | 59 | 0.168 | 0.059 |
| Combination 21  Age, ATP, ACh, Nitrite, MCP-1, IL-5 | 56 | 40 | 61 | 73 | 0.149 | 0.779 |
| Combination 22  ATP, ACh, NO, Nitrite, MCP-1, IL-5 | 56 | 40 | 61 | 68 | 0.381 | 1.000 |
| Combination 23  Gender, Age, Volume, ATP, ACh, NO,  Nitrite, MCP-1, IL-5 | 34 | 62 | 65 | 77 | 0.146 | 0.698 |
| n= Number of participants included in the analysis; e= Number of participants excluded in the analysis due to missing data; Null model= Model with no predicting variable(s), just the intercept; Pr Null model (%)= Percentage of cases for which the dependent variable was correctly predicted given the null model; New model= Model with predicting variable(s); Pr New model (%)= Percentage of cases for which the dependent variable was correctly predicted given the new model; O test= Omnibus Test of Model Coefficients; HL test= Hosmer-Lemeshow goodness of fit test. Bold values represent statistically significant p-value of ≤0.05.  *All the urinary biomarker values were normalised to urinary creatinine concentrations. | | | | | | |

**Table S4 – Standard errors of the constant and the coefficients of variables used in the OAB prediction equation in Table 3.**

| **Predictive model** | **Coefficient** | **SE** |
| --- | --- | --- |
| Combination 17 |  |  |
| Age | 5.393 | 2.256 |
| Gender | 1.797 | 0.717 |
| IL-5/Cr | 34.767 | 56.331 |
| ATP/Cr | -562.743 | 629.316 |
| Constant | -3.090 | 1.200 |
| SE= Standard error; Cr= Creatinine, all the urinary biomarker values were normalised to urinary creatinine concentrations. | | |
